# Prevalence and associated factors of female-perpetrated intimate partner violence against men in Africa: a systematic review and meta-analysis

**DOI:** 10.1186/s12889-026-26936-x

**Published:** 2026-03-10

**Authors:** Subah Abderehim Yesuf, Sisay Seyfe Demeke, Abel Yirga Berhe, Yasir Mohammed Zaroug Elradi, Natnael Belay Tenaw

**Affiliations:** 1 Department of Family Medicine, St. Peter’s Specialized Hospital, Addis Ababa, Ethiopia; 2 Department of Family Medicine, St. Paul’s Hospital Millennium Medical College, Addis Ababa, Ethiopia; 3 Department of Biochemistry, Hawassa University, Hawassa, Ethiopia; 4 Internal Medicine Department, Ethio-Istanbul General Hospital, Addis Ababa, Ethiopia; 5 Warwick Medical School, University of Warwick, Coventry, UK; 6 Dhaman Health Company, Kuwait City, Kuwait; 7 Eka Kotebe General Hospital, Addis Ababa, Ethiopia

**Keywords:** Intimate partner violence, IPV, Men, Africa, Prevalence, Partner controlling behavior, Meta-analysis

## Abstract

**Background:**

Intimate partner violence (IPV) is a critical public health issue traditionally recognized as primarily affecting women; however, men are also victims of IPV, experiencing various forms of violence from their female intimate partners. In Africa, where patriarchal norms prevail, research on IPV against men remains limited and fragmented. This systematic review and meta-analysis aimed to estimate the pooled prevalence of IPV against men across African countries and identify associated risk factors.

**Methods:**

A comprehensive literature search was conducted across multiple databases including PubMed, Epistemonikos, Cochrane Library, Africa Index Medicus, and African Journals Online through November 5, 2025. Cross-sectional studies reporting on IPV prevalence and associated factors among men in Africa were included. Data extraction and quality assessment followed standardized protocols, and pooled prevalence was estimated using a random-effects meta-analysis with Stata version 17. Heterogeneity was assessed using the I² statistic, and publication bias was evaluated via funnel plots and Egger’s test.

**Results:**

Sixteen studies encompassing 17,939 men from six African countries met the inclusion criteria. The pooled prevalence of IPV against men was 40.49% (95% CI: 31.53%–49.44%), with psychological violence being the most prevalent subtype. Partner controlling behavior (OR = 5.02, 95% CI: 3.35–7.53), partner alcohol use (OR = 1.94, 95% CI: 1.61–2.34), history of IPV perpetration (OR = 4.21, 95% CI: 2.37–7.48), exposure to parental IPV in childhood (OR = 1.64, 95% CI: 1.34–1.98), marital separation (OR = 2.01, 95% CI: 1.21–3.33), and having one wife (OR = 0.50, 95% CI: 0.36–0.68) were significantly associated with increased odds of IPV victimization.

**Conclusions:**

Nearly two out of five men in Africa experience IPV, a considerably high prevalence that underscores this overlooked public health concern. Partner controlling behavior emerges as a strong predictor of IPV against men, highlighting the need for gender-inclusive prevention strategies that address controlling dynamics within relationships. These findings advocate for expanded IPV screening, tailored interventions, and policies that promote awareness and support for male victims across diverse African contexts.

**Supplementary Information:**

The online version contains supplementary material available at 10.1186/s12889-026-26936-x.

## Background

Intimate partner violence (IPV) is a pervasive public health issue characterized by any behavior within an intimate relationship that causes physical, psychological, or sexual harm to those in that relationship [1]. Although it is recognized primarily for its impact on women, growing evidence indicates that men, apart from being culprits, are also victims of IPV, experiencing all forms of violence by intimate partners [2, 3]. Since 2013, the World Health Organization has acknowledged IPV against men as an important public health issue [4].

The prevalence of IPV against men varies widely across settings, with reported estimates ranging from 3.4% to 76.1% depending on the population studied and the types of violence assessed [5, 6]. In Africa, where gender inequality is conceived to prevail within predominantly patriarchal and male-dominated cultures, only a few studies have investigated female-perpetrated IPV against men [7, 8]. Consequently, the concept of female-perpetrated IPV against men is often met with skepticism, which further compounds the already low rates of care-seeking behavior among men experiencing IPV [6, 9, 10].

Despite increasing recognition of IPV as a critical public health issue on both genders, research has predominantly focused on female victims, leaving significant gaps in characterizing female-perpetrated IPV experiences among men, compounded by anticipated underreporting, particularly within the African context [10]. Understanding the magnitude and patterns of IPV against men in Africa is critical for informing gender-inclusive, nuanced violence prevention and support services.

While systematic reviews and meta-analyses on IPV in Africa exist, along with empirical studies examining IPV against men in individual African countries, no comprehensive synthesis has quantitatively pooled prevalence data specifically on male IPV victimization across the continent. Previous reviews have primarily focused on female victims, sub-Saharan Africa specifically, lacking continent-wide meta-analytic synthesis of prevalence and associated factors among men.

Therefore, this study aims to fill this gap by conducting a systematic review and meta-analysis to estimate the pooled prevalence of female-perpetrated IPV against men and examine its associated factors in African countries. By collating the available evidence, this review will provide valuable insights to guide policy, research, and intervention programs addressing IPV among men in the continent.

## Methods

### Protocol and registration

**Fig. 1:**
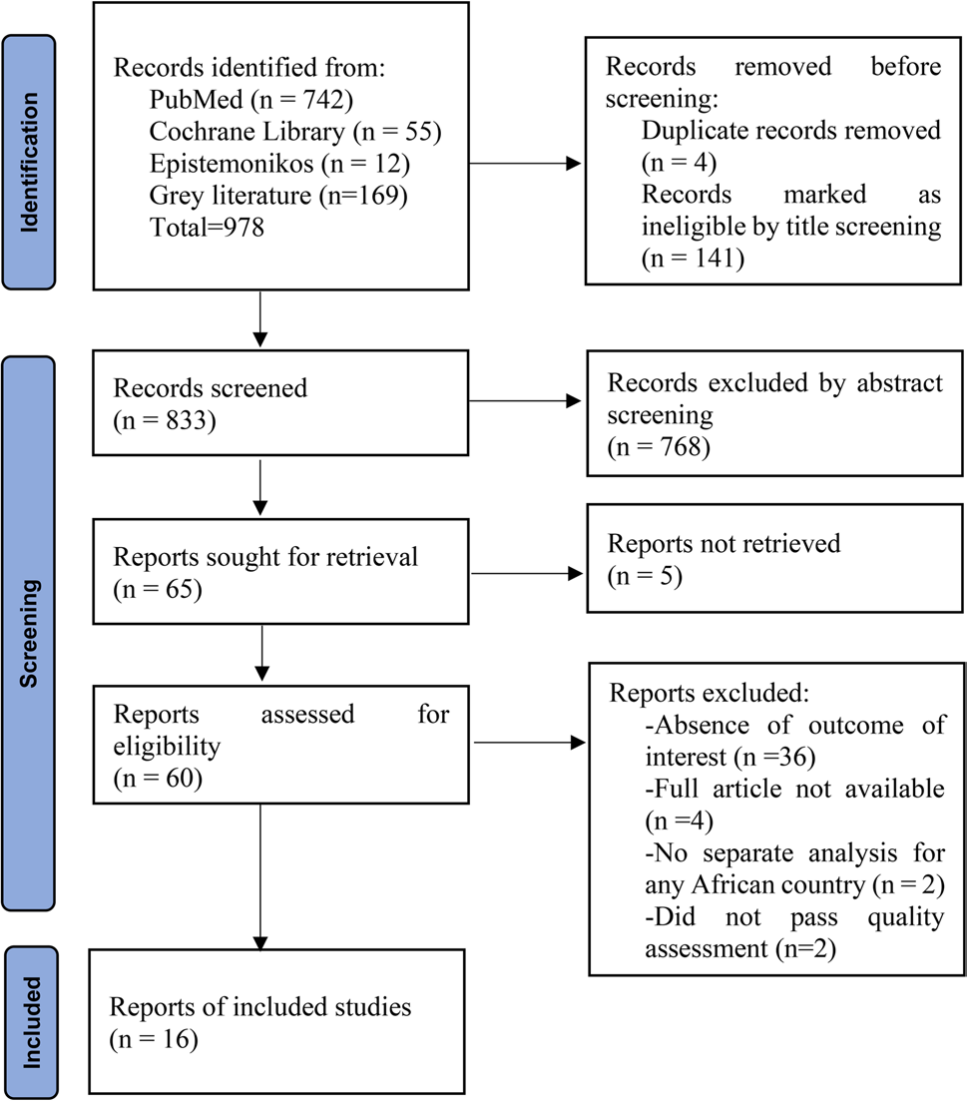
PRISMA flow diagram of articles screening and process of selection

The current systematic review and meta-analysis was undertaken in compliance with Preferred Reporting Items for Systematic Reviews and Meta-Analyses (PRISMA) guidelines [11] (Fig. 1). The review protocol was prospectively registered in PROSPERO under the registration number CRD420251177603.

### Eligibility criteria

#### Inclusion criteria

The following eligibility criteria were applied using the PCC framework (Population: adult men in Africa; Concept: IPV victimization; Context: any setting):

- Quantitative studies reporting the prevalence or magnitude of female-perpetrated IPV against adult men (≥ 18 years) in any African country.
- Studies examining IPV against men in heterosexual relationships (current or former female partners).
- Published peer-reviewed articles, grey literature, conference abstracts, theses/dissertations, and unpublished reports of any study design (cross-sectional, cohort, case-control).
- Articles using any validated or study-defined IPV measurement method, without restrictions on specific diagnostic instruments.
- Publications from any time period up to the search date (November 5, 2025).
- Articles published in English (or with English abstracts available for screening).

#### Exclusion criteria

- Case studies, panel discussions, editorials, commentaries, and opinion pieces.
- Studies that could not be accessed after two email attempts to the corresponding author.
- Studies not published in English.
- Studies conducted on non-human subjects.
- Qualitative studies.
- Studies lacking sufficient data to compute the prevalence of IPV against men.

### Search strategy and information sources

A search strategy was implemented using electronic databases, namely PubMed, Epistemonikos, Cochrane Library, Africa Index Medicus, and African Journals Online, to identify peer-reviewed and English-language articles published until November 5, 2025.

The search strategy employed Medical Subject Headings (MeSH), free-text keywords, Boolean operators, truncation, and phrase searching, adapted for each database. PubMed served as the primary database, with database-specific terms applied to others (full strings: Supplementary File 1).

Reference lists of included studies and relevant reviews were hand-searched. Additionally, Google Scholar (first 200 results per search string) and Google were searched for supplementary records such as recent theses, conference papers, and preprints. No existing systematic reviews or protocols specifically addressing prevalence of IPV against men across Africa were identified.

Grey literature and unpublished studies were systematically sought through OpenGrey, ProQuest Dissertations & Theses Global, author contacts, and professional networks such as ResearchGate. This approach yielded two studies included in the synthesis.

### Study selection

All identified records were imported into Mendeley Desktop version 1.19.8 reference management software, and duplicates were removed. Two independent reviewers (SAY and SSD) conducted an initial screening of metadata, that is, titles, keywords and abstracts against the predefined inclusion and exclusion criteria. Studies that clearly did not meet the eligibility criteria were excluded at this stage. Full texts of potentially eligible articles were then retrieved and independently assessed for inclusion by the same reviewers. Discrepancies at any stage were resolved through discussion, and if consensus was not reached, a third reviewer (AYB) was consulted. Reasons for exclusion at the full-text screening stage were documented. The overall study selection process was illustrated using a PRISMA flow diagram, detailing the numbers of records identified, screened, eligible, and included in the review (Fig. 1).

### Data extraction and management

Data extraction was conducted using a standardized and piloted data collection form to ensure consistency and accuracy. Two independent reviewers (SAY and NBT) extracted relevant data from all eligible studies, including author information, year of publication, country of study, sample size, number of IPV cases, prevalence estimates, type of IPV, and associated covariates (Supplementary Table S1). Discrepancies between reviewers were resolved through discussion and consultation with a third reviewer (YMZE) when necessary. Extracted data were entered into a dedicated database for further analysis. The process was conducted in duplicate to minimize errors and reduce potential bias. All data management activities were performed using secure electronic software to maintain data integrity and facilitate subsequent quantitative synthesis.

### Quality assessment

The methodological quality of each included study was independently assessed by two reviewers using the Joanna Briggs Institute (JBI) Critical Appraisal Checklist for Prevalence Studies, designed specifically for evaluating bias and methodological rigor in observational prevalence studies [12]. This checklist assesses domains such as sample representativeness, appropriateness of recruitment, sample size, description of study subjects and setting, data collection methods, reliability and validity of outcome measurement, and appropriateness of statistical analysis.

Each item was rated as “Yes,” “No,” or “Unclear,” and disagreements between reviewers were resolved through discussion or adjudication by a third reviewer when necessary. Studies were classified as having low, moderate, or high risk of bias based on their total appraisal scores. Only studies with low or moderate risk of bias were included in the meta-analyses to enhance the validity of the pooled estimates. One study failing quality standards was excluded from synthesis. Additionally, one study from predatory publishers (per Beall’s List) was excluded due to unreliable peer review (Fig. 1).

### Outcome measurements

IPV against men was operationalized per WHO multicountry study definitions as any act of physical, sexual, psychological, or economic violence perpetrated by a current or former female intimate partner. The primary outcome of this review was to estimate the pooled prevalence of IPV against men in African countries. Specific aspects measured across included studies encompassed:

- Physical violence: Slapping, hitting, kicking, choking, use of weapons. Sexual violence: Forced intercourse, coerced sexual acts.
- Psychological violence: Insults, humiliation, threats, controlling behaviors.
- Economic violence: Controlling access to financial resources, preventing work.

### Data synthesis and statistical analysis

Data synthesis was performed using STATA version 17. The primary outcome, the pooled prevalence of IPV against men, was estimated using a random-effects meta-analysis model due to observed heterogeneity among the studies.

Heterogeneity among studies, a critical issue in meta-analysis, was assessed using the I² statistic and Cochran’s Q test, which tests the null hypothesis that all studies share a common effect [13]. An I² value greater than 75% and a p-value less than 0.05 from the Q test were considered indicative of substantial heterogeneity. Where substantial heterogeneity was present, subgroup and sensitivity analyses were conducted to explore potential sources based on study country.

Since significant heterogeneity was observed (I² = 99.39%, *p* < 0.001), the use of a random-effects model was warranted. To account for between-study variance, the Restricted maximum–likelihood method was applied in the random-effects meta-analysis [14]. Publication bias was evaluated visually using funnel plots and quantitatively with Egger’s regression test. A p-value less than 0.05 was used as a threshold to indicate significant publication bias. Moreover, where meta-analysis was not feasible due to insufficient or highly heterogeneous data, a narrative synthesis was performed to summarize and interpret the findings systematically. 

### Ethics approval and consent to participate

This study is a systematic review and meta-analysis that exclusively utilizes data from previously published articles accessed through publicly available databases. Therefore, ethical approval from an Institutional Review Board was not required.

## Results

### Study characteristics

The articles included in this meta-analysis were all cross-sectional in design. Of the sixteen studies, seven were conducted in Uganda, four in Nigeria, two in Kenya, and one each in Tanzania, South Africa, and Rwanda, collectively encompassing 17,939 ever-partnered men. Publication years ranged from 2003 to 2025, with sample sizes varying widely from 121 to 5109 male participants (Table 1).

**Table 1:** Characteristics of the studies included to assess the pooled prevalence of intimate partner violence against men in Africa

First Author	Year of publication	Sample size	IPV prevalence	Type of IPV		
				Psychological	Physical	Sexual
Ashimi et al. [15]	2023	322	45.0	44.4	23.3	15.8
Waila et al. [16]	2022	2858	43.6	36.9	20.2	8.2
Amole et al. [17]	2015	302	66.8	47.7	45.7	9.6
Gass et al. [18]	2011	641	20.9	NR	NR	NR
Ringwald et al. [19]	2023	1321	20.0	18.0	7.0	4.0
Gubi et al. [20]	2022	2559	44.0	36.0	20.0	8.0
Maposa et al. [21]	2023	1371	18.4	16.7	8.7	1.1
Odemba et al. ^£^[22]	2025	398	76.1	47.5	12.2	16.5
Ogbonnaya et al. [23]	2020	121	65.3	62.8	21.5	7.4
Ohurira et al. [24]	2023	387	50.4	NR	NR	NR
Kitutu et al. [25]	2025	397	47.4	34.5	7.2	10.3
Kinyanda et al. [26]	2016	416	41.8	39.3	21.5	5.4
Igwe et al. ^€^ [27]	2020	122	36.9	16.4	3.3	10.6
Mulawa et al. [28]	2018	1113	34.8	29.2	8.4	11.1
Koenig et al. [29]	2003	5109	19.8	18.7	3.0	NR
Oseni et al. [30]	2022	502	18.7	11.6	12.7	9.8

*NR* Not reported

^£ and €^: reported economic violence of 23.8% and 2.4%, respectively

The reported prevalence of IPV against men showed substantial variation across studies, ranging from a low of 18.4% to a high of 76.1%. Psychological IPV was generally more prevalent than physical and sexual forms in most studies. For example, Amole et al. (2015) reported a psychological IPV prevalence of 66.8%, alongside physical and sexual IPV prevalences of 47.7% and 9.6%, respectively. In contrast, Maposa et al. (2023) documented lower corresponding prevalences of 16.7%, 8.7%, and 1.1%, respectively (Table 1).

Psychological IPV prevalence generally exceeded physical and sexual IPV in most studies. For instance, Amole et al. (2015) reported a psychological IPV prevalence of 66.8%, with physical and sexual IPV at 47.7% and 9.6%, respectively, while Maposa et al. (2023) documented lower prevalences of 16.7%, 8.7%, and 1.1%, respectively. While some studies did not report subtype-specific IPV data, economic violence was reported in only two studies (Odemba et al. and Igwe et al.) at 23.8% and 2.4%, respectively (Table 1).

### Prevalence of intimate partner violence against men

**Fig. 2:**
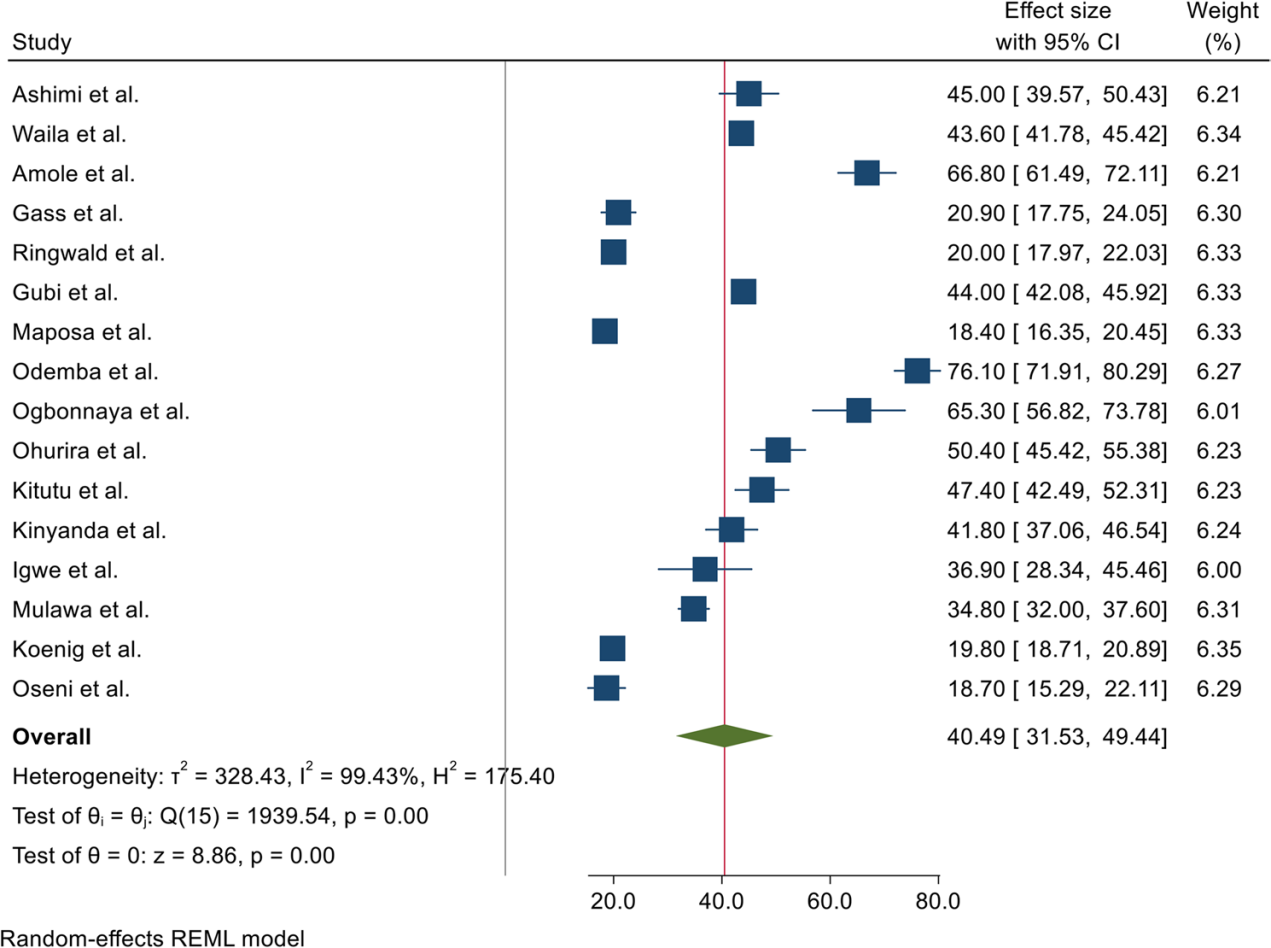
Meta-analysis forest plot of pooled prevalence of IPV against men in Africa, 2025

The forest plot illustrates significant variation in IPV prevalence across individual studies, attributable to differences in geographic region, population characteristics, and IPV measurement methods. Due to substantial heterogeneity among the studies (I² = 99.43%, p < 0.001), a random-effects model was utilized to compute the overall pooled prevalence. The combined estimate reveals that approximately 40.49% (95% CI: 31.53% to 49.44%) of men in Africa experience IPV, highlighting a considerable public health burden (Figure 2).

**Fig. 3:**
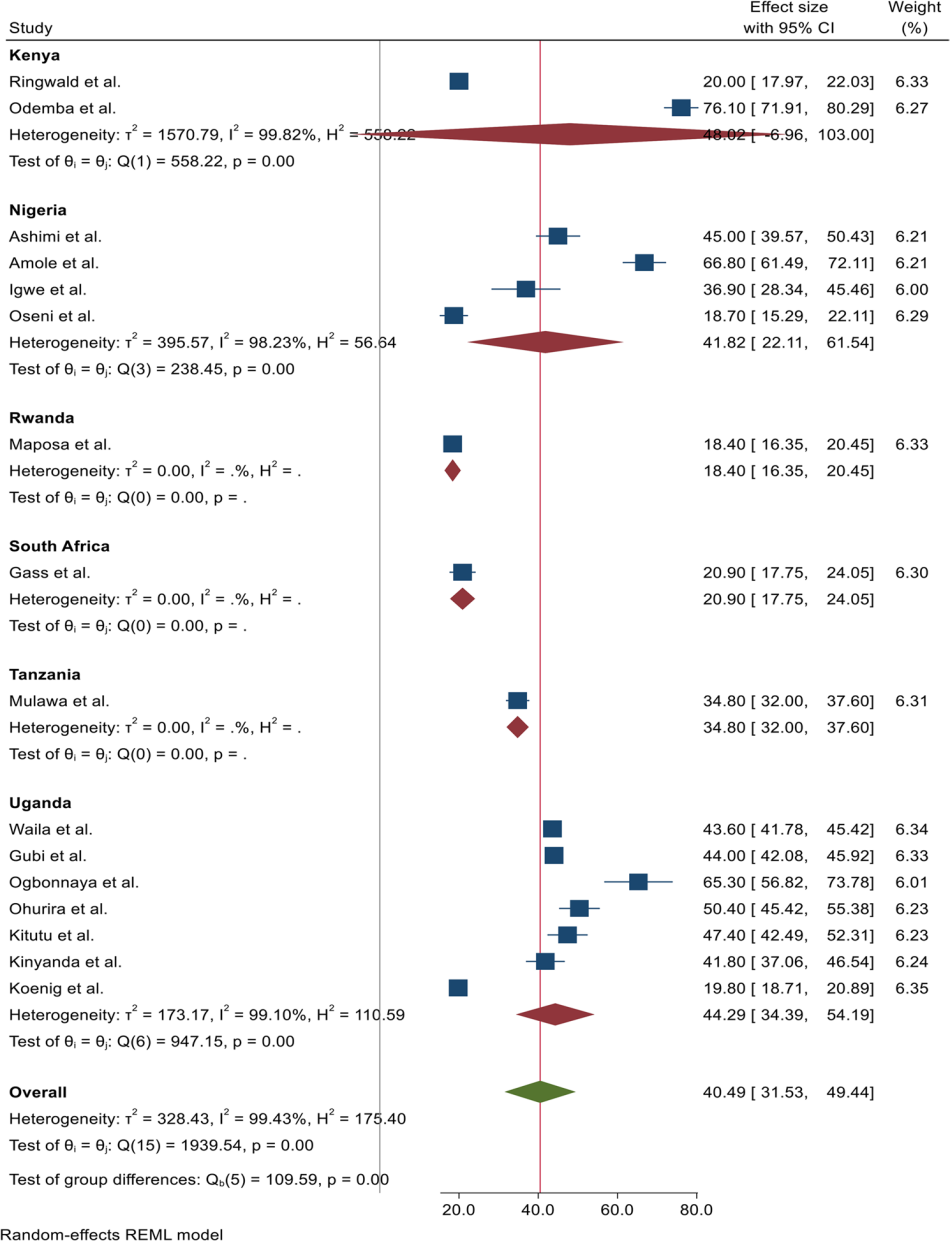
Sub-group meta-analysis of intimate partner violence against men in Africa, 2025

Subgroup analyses further explored variations by region, providing deeper insights into the patterns of IPV victimization among men across the continent. The estimated pooled prevalence was highest in Kenya at 48.69% (95% CI: 6.96–103.00), and lowest in Rwanda at 18.40% (95% CI: 16.35–20.45) (Figure 3).

### Publication bias and sensitivity analysis 

**Fig. 4:**
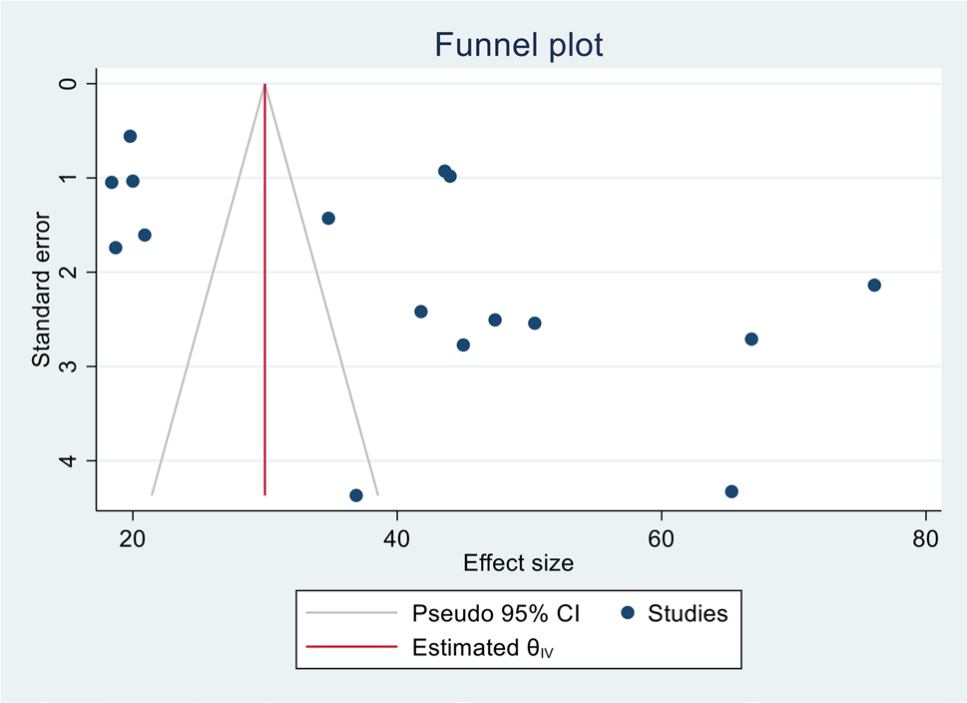
Funnel plot for publication bias, proportion represented in the x-axis and standard error of proportion on y-axis

**Fig. 5:**
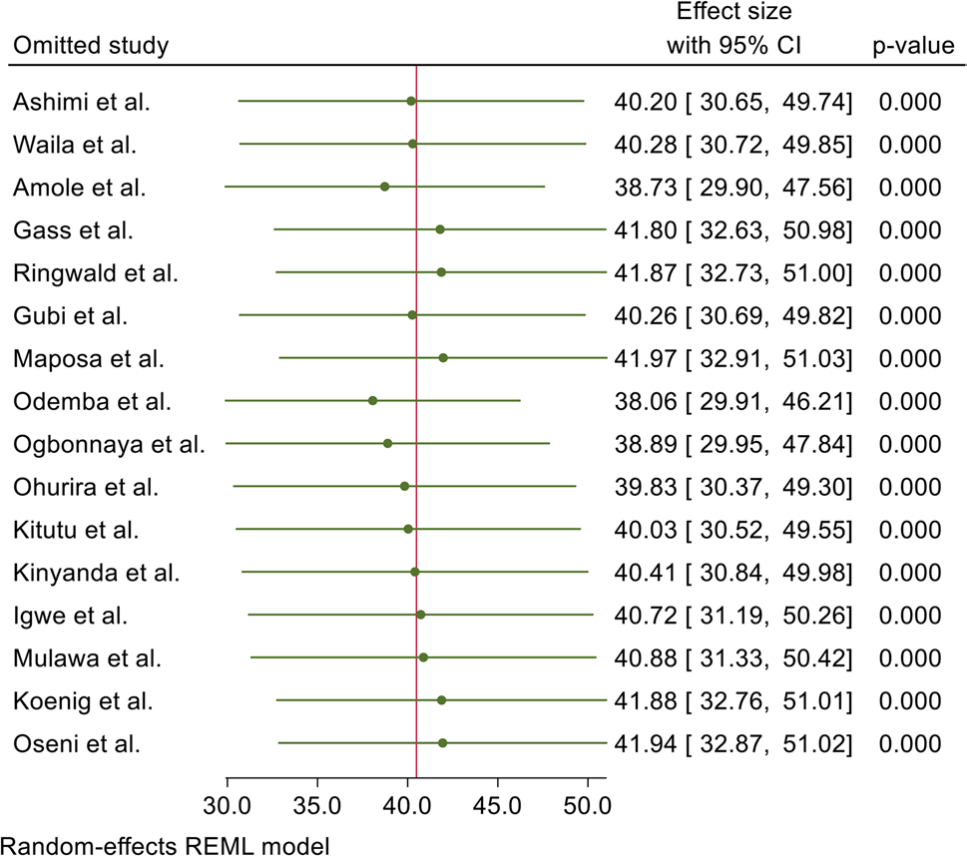
Leave-one-out meta-analysis of intimate partner violence against men in Africa, 2025

Egger’s regression test and a funnel plot were used to assess publication bias. Subjectively, the funnel plot showed a skewed distribution, suggesting the presence of publication bias (Figure 4). Furthermore, Egger’s regression test yielded an objective p-value of 0.0184, confirming the presence of publication bias. A leave-one-out sensitivity analysis was conducted to assess the influence of individual studies on the overall outcome. With the Restricted maximum–likelihood model, it indicated that the results were robust, as the point estimate after omitting each study remained within the confidence interval of the combined analysis, and there was no substantial change in overall heterogeneity (Figure 5).

### Univariate meta-regression to explore factors contributing to heterogeneity

In this section, univariate meta-regression was conducted to identify factors contributing to heterogeneity in IPV against men. Geographic region, sample size, and year of publication were examined, but none significantly influenced heterogeneity (Table 2). However, subgroup analysis by region was performed based on a priori theoretical rationale.

**Table 2:** Univariate meta-regression to explore factors contributing to heterogeneity in IPV against men in Africa

Factor	Coefficient	P-value	95% Confidence interval
Geographic region	0.193	0.920	-3.553,3.938
Sample size	-0.005	0.114	-0.012,0.001
Year of publication	0.987	0.212	-0.565,2.539

### Factors associated with IPV

**Fig. 6:**
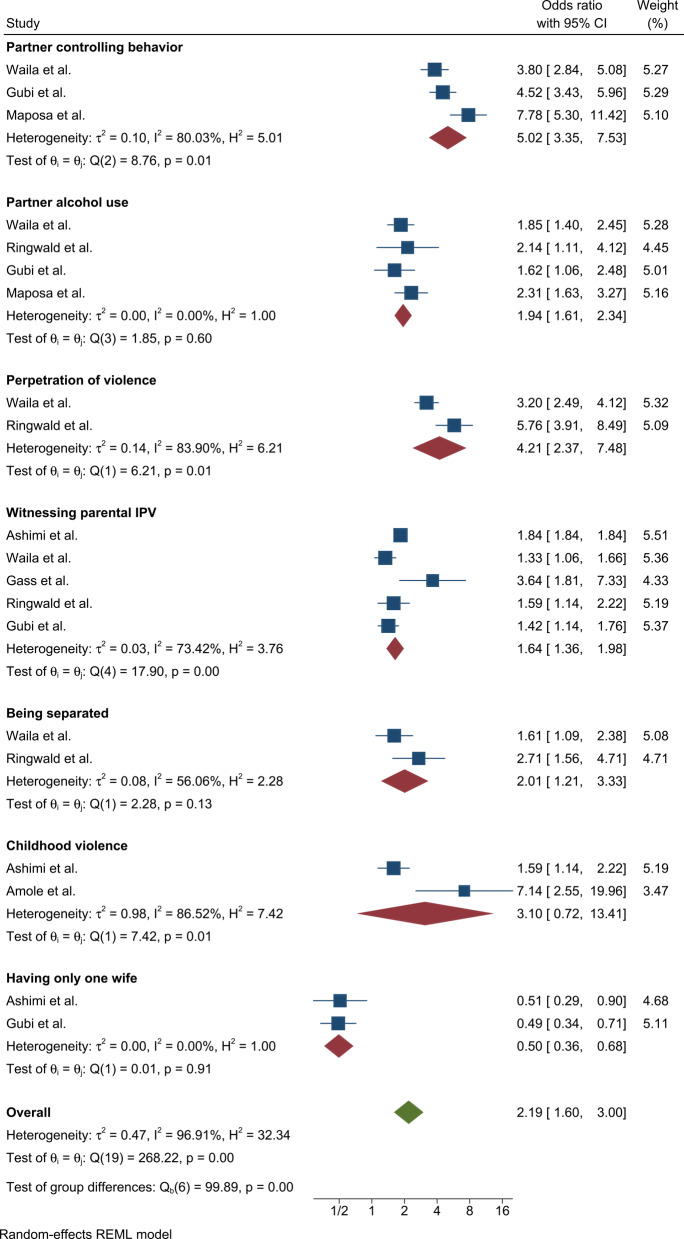
Forest plot of factors associated with IPV among men in Africa, 2025

Among the 16 included studies, three studies reported an association between partner controlling behavior and IPV. The pooled odds ratio (OR) from these studies was 5.02 (95% CI: 3.35–7.53), indicating that men with controlling partners were significantly more likely to experience IPV compared to their counterparts (Figure 6).

Four studies reported an association between IPV and partner alcohol use. The pooled odds ratio indicated a statistically significant association between partner alcohol use and IPV victimization (OR = 1.94, 95% CI: 1.61–2.34) (Figure 6).

Two studies identified an association between IPV victimization and perpetration of violence. The pooled odds ratio indicated a statistically significant association between IPV perpetration and IPV victimization (OR = 4.21, 95% CI: 2.37–7.48; Figure 6).

Five studies reported an association between IPV victimization among men and exposure to parental IPV during childhood. The pooled odds ratio showed a statistically significant association between childhood exposure to parental IPV and subsequent IPV victimization (OR = 1.64, 95% CI: 1.34–1.98; Figure 6).

Two studies reported an association between marital separation/divorce status and IPV victimization among men. The pooled odds ratio demonstrated a statistically significant association between being separated or divorced and IPV victimization (OR = 2.01, 95% CI: 1.21–3.33; Figure 6).

Two studies identified an association between IPV victimization among men and prior exposure to childhood violence. The pooled odds ratio revealed a statistically significant association between childhood violence exposure and IPV victimization (OR = 3.10, 95% CI: 0.72–13.41; Figure 6).

Additionally, two studies documented an association between IPV victimization and monogamous marital status among men. The pooled odds ratio confirmed a statistically significant association between having only one wife and lower IPV victimization risk (OR = 0.50, 95% CI: 0.36–0.68; Figure 6).

Factors identified in only a single study include the richest wealth level (AOR: 0.54, 95% CI: 0.30–0.94) [21], having ≥5 children (AOR: 3.93, 95% CI: 1.16–13.29) [17], being childless (AOR: 7.32, 95% CI: 1.70–35.20) [15], having no income (AOR: 3.93, 95% CI: 1.40–11.00) [18], residing in the Western region (AOR: 1.41, 95% CI: 1.03–1.93) [20], and fear of a partner most of the time (AOR: 5.10, 95% CI: 2.91–8.96) [16].

## Discussion

Owing to the paucity of data pertaining to IPV against men and recognizing that men are not immune to the spectrum of IPV experiences, this systematic review and meta-analysis was conducted to synthesize evidence from studies conducted across six African countries. The overall pooled prevalence of female-perpetrated IPV was notably high, with approximately 40.49% (95% CI: 31.53% to 49.44%) of men in Africa reporting experiences of IPV. This finding reveals the significant, yet often under-recognized, burden of IPV among men on the continent.

The pooled female-perpetrated IPV prevalence estimated in this study is notably higher than many prevalence rates reported from studies conducted outside Africa. This finding aligns with a global report documenting pooled prevalences of psychological IPV at 44%, physical IPV at 20%, and sexual IPV at 7% among men across various settings [31]. It is also supported by data from Cameroon (psychological IPV: 26.5%, physical: 24.4%, sexual: 2.3%) and Sierra Leone (psychological: 23.4%, physical: 14.9%, sexual: 2.7%) [32].

However, the IPV prevalence in this study differs from rates observed in mainland China and Portugal, where the corresponding prevalence of IPV victimization among men was reported as 8% and 62.5%, respectively [33, 34]. Similarly, European epidemiological research showed IPV prevalence among men closer to 19.8%, with variations across countries but generally lower prevalence compared to the African pooled estimate [35]. Additionally, the present finding is higher than the trend observed in Rwanda, which showed a decrement in prevalence from 21% in 2015 to 18% in 2020 in men [36].

Of particular note, the relatively high prevalence in this meta-analysis may be partially explained by the inclusion of studies conducted during the COVID-19 pandemic [16, 23, 27, 30], a period associated with increased IPV due to heightened stress, anger, frustration, and social isolation [37]. 

These differences may partly reflect diverse sociocultural norms, levels of gender inequality, reporting practices, and the varying emphasis on recognizing male victimization. Patriarchal social structures prevalent in many African societies may simultaneously contribute to higher IPV rates and underreporting due to stigma. Additionally, methodological variation across studies also sampling, definitions of IPV, and measurement tools can influence prevalence estimates. The elevated prevalence in African contexts may be influenced by intersecting factors such as distinct and stigma affecting disclosure rates.

The wide confidence interval and evidence of high heterogeneity across the included studies reflect variations in cultural, regional, and methodological contexts influencing IPV dynamics, emphasizing the need for context-specific research and interventions. Given these observations, the findings should be interpreted with caution; nevertheless, they provide a compelling evidence base for health policymakers, researchers, and practitioners to recognize and prioritize IPV against men within the broader violence prevention agenda in Africa. Promoting gender-inclusive approaches to violence reduction and health promotion is essential, as IPV against men can serve as a proxy indicator for IPV against their partners, indicating the intertwined nature of intimate partner relationships in the context of violence [34, 38].

The analysis identified partner controlling behavior as increasing IPV victimization risk among men. This finding aligns with evidence from the literature highlighting controlling behavior as a significant risk factor for IPV [39, 40]. Partner controlling behaviors, such as monitoring movements, limiting contact with others, and exerting power over decisions, create a dynamic of coercive control that increases vulnerability to various forms of IPV.

In this analysis, partner alcohol use was associated with increased IPV victimization odds. This is supported by several previous works [41–43]. This can be justified by the fact that alcohol can lead to a decrease in judgment and an increase in aggression, which are factors that can contribute to IPV [44].

Interestingly, this study highlights that men with a history of IPV perpetration were four times more likely to experience IPV victimization themselves. This co-occurrence of victimization and perpetration reflects a pattern of bidirectional violence within intimate relationships. Such findings are consistent with studies from China and Portugal, which have documented that men who perpetrate IPV are also at increased risk of victimization, highlighting the complex and reciprocal dynamics of IPV in some relationships [33, 34]. Importantly, this dynamic suggests the need to address IPV with consideration of mutual violence patterns rather than unidirectional models alone.

Moreover, this review highlighted that exposure to parental IPV during childhood was associated with higher odds of IPV victimization. This finding is consistent with previous reports, which independently documented that exposure to parental violence is positively associated with IPV victimization later in life [45, 46]. Such exposure has been shown to contribute to behavioral, emotional, and social difficulties and may perpetuate intergenerational cycles of violence by normalizing violence within intimate relationships [47].

In this review, men who were separated or divorced experienced higher odds of IPV victimization compared to those in ongoing partnerships. This finding is in concordance with existing evidence documenting an association between IPV victimization and separated/divorced status. The observed association likely reflects the dynamic nature of IPV during relationship dissolution, stemming from prior IPV that contributed to relationship breakdown [48, 49]. 

## Strengths and limitations

This meta-analysis has several notable strengths. It provides a comprehensive synthesis covering six African countries, enhancing the generalizability of the findings across diverse settings. The inclusion of a substantial number of studies with a large pooled sample size yields robust and precise prevalence estimates for female-perpetrated IPV against men, a population often neglected in IPV research. The study employed rigorous and transparent methods, including strict adherence to PRISMA guidelines, systematic data extraction, critical appraisal based on Joanna Briggs Institute criteria, and random-effects meta-analysis models, bolstering the reliability and validity of the findings. Sensitivity and subgroup analyses further strengthened the conclusions by addressing between-study heterogeneity and contextual variations.

However, limitations exist. First, all included studies were cross-sectional and observational, precluding causal inference; pooled estimates thus represent summaries of reported associations rather between identified factors and IPV than causal risk indicators. Second, considerable heterogeneity across populations, IPV definitions, measurement instruments, and cultural contexts may impact the comparability of results and pooled prevalence, despite statistical adjustments. Third, the review could not capture data on IPV intensity, chronicity, or severity, which are critical to understanding the full spectrum of IPV experiences. Additionally, the inconsistent reporting of economic violence and other IPV subtypes across studies limits comprehensive synthesis. Finally, potential underreporting due to stigma among male victims, detected publication bias, and geographical gaps from under-represented African regions may underestimate prevalence and limit generalizability.

## Conclusions

Nearly two out of five men in Africa report experiencing female-perpetrated IPV, underscoring an under-recognized public health issue that necessitates gender-inclusive strategies. Policymakers should incorporate opportunistic screening for male IPV victimization into primary health services to enable early detection and context-appropriate support. Community-based interventions are recommended to address intergenerational violence cycles via family-centered education and tailored counseling, targeting childhood exposure to parental IPV. Future research should prioritize methodologically robust longitudinal studies to clarify temporal associations and causality, addressing limitations of cross-sectional designs. Together, these measures can guide context-specific prevention efforts across African settings.

## Supplementary Information

Supplementary Material 1.

Supplementary Material 2.

Supplementary Material 3.

## Data Availability

All data generated or analyzed during this study are included in this article.

## Abbreviations

IPV: Intimate partner violence
MeSH: Medical Subject Headings
PRISMA: Preferred Reporting Items for Systematic Reviews and Meta-Analyses
