# Supplementary material for: Prevalence and associated factors of female-perpetrated intimate partner violence against men in Africa: a systematic review and meta-analysis
Source: BMC Public Health. 2026 Mar 10;26:1258. doi: 10.1186/s12889-026-26936-x (PMC13088386; doi:10.1186/s12889-026-26936-x)
Supplement: Supplementary file 1 — Supplementary Material 1. [file 12889_2026_26936_MOESM1_ESM.docx]

**Search syntax (PubMed)**

("Intimate Partner Violence"[MeSH] OR "Domestic Violence"[MeSH] OR "intimate partner violence" OR "domestic violence" OR IPV) AND ("Men"[MeSH] OR "men"[MeSH] OR men OR male OR males) AND ("Africa"[MeSH] OR Africa OR "Sub-Saharan Africa" OR "East Africa" OR "West Africa" OR "Southern Africa" OR "Central Africa") AND ("Prevalence Studies"[MeSH] OR prevalence OR magnitude OR epidemiology OR incidence) AND ("Risk Factors"[MeSH] OR "risk factors" OR determinants OR correlates)

Note: Search strings were adapted using database-specific subject headings, field labels, and syntax for Epistemonikos, Cochrane Library, Africa Index Medicus, and African Journals Online. Database-specific adaptations are available upon request.
