# Supplementary material for: Prevalence and associated factors of female-perpetrated intimate partner violence against men in Africa: a systematic review and meta-analysis
Source: BMC Public Health. 2026 Mar 10;26:1258. doi: 10.1186/s12889-026-26936-x (PMC13088386; doi:10.1186/s12889-026-26936-x)
Supplement: Supplementary file 2 — Supplementary Material 2. [file 12889_2026_26936_MOESM2_ESM.docx]

## Grey Literature Search Results

- Grey literature databases: OpenGrey (n=9 records)
- Theses and dissertations: ProQuest Dissertations & Theses Global (n=12)
- Google Scholar (first 200 results per search string, n=128 records)
- Author contacts: n=4
- ResearchGate (n=16 records)

**Total grey literature records identified: n=169**
